# Addressing Challenges in Health Technology Assessment Institutionalization for Furtherance of Universal Health Coverage Through South-South Knowledge Exchange: Lessons From Bhutan, Kenya, Thailand, and Zambia

**DOI:** 10.1016/j.vhri.2020.12.011

**Published:** 2021-05

**Authors:** Taeyoung Kim, Manushi Sharma, Yot Teerawattananon, Cecilia Oh, Leslie Ong, Peter Hangoma, Deepika Adhikari, Pempa Pempa, Angela Kairu, Stacey Orangi, Saudamini Vishwanath Dabak

**Affiliations:** 1 Health Intervention and Technology Assessment Program, Nonthaburi, Thailand; 2 Saw Swee Hock School of Public Health, National University of Singapore, Singapore; 3 HIV, Health and Development Team, United Nations Development Programme, Bangkok, Thailand; 4 Department of Health Policy and Management, University of Zambia, Lusaka, Zambia; 5 Essential Medicines and Technology Division, Department of Medical Services, Ministry of Health, Thimphu, Bhutan; 6 Health Economics Research Unit, KEMRI-Wellcome Trust Research Programme, Nairobi, Kenya

**Keywords:** health technology assessment, low- and middle-income countries, public health policy, South-South knowledge exchange, universal health coverage

## Abstract

Health Technology Assessment (HTA), a tool for priority setting, has emerged as a means of ensuring the sustainability of a Universal Health Coverage (UHC) system. However, setting up an effective HTA system poses multiple challenges and knowledge exchange can play a crucial role in helping countries achieve their UHC targets. This article reports the results of the discussion during a preconference session at the 2019 HTAsiaLink Conference, an annual gathering of HTA agencies in Asia, which supports knowledge transfer and exchange among HTA practitioners. As part of this discourse, 3 main HTA challenges were identified based on experiences of selected countries in Asia and Africa, namely Bhutan, Kenya, Thailand, and Zambia: availability of funding, building technical capacity, and achieving buy-in among stakeholders for successful translation of HTA research into UHC policy. The potential solutions identified through this South-South engagement included establishing a legal mandate for HTA, building local technical capacity through partnerships and enhancing strategic communication with stakeholders to increase awareness, among others. South-South Knowledge Exchange can therefore be instrumental in sharing lessons learned from common challenges and offer potential solutions to address capacity building initiatives for HTA in LMICs.

## Highlights

• Health technology assessment is an effective tool for priority setting for countries seeking to achieve Universal Health Coverage.
• Three of the main challenges faced by countries in institutionalizing Health Technology Assessment are limited funding, lack of technical capacity, and stakeholder engagement.
• South-South knowledge exchange is a useful means of learning from peers to address challenges.

## Introduction

An increasing number of low- and middle-income countries (LMICs) are moving toward providing Universal Health Coverage (UHC) as part of their efforts to achieve the Sustainable Development Goals by 2030 (SDGs).^1^ To ensure sustainability of the UHC agenda, there is need for evidence-informed priority setting of key interventions.^2^^,^^3^ Health Technology Assessment (HTA), a tool for priority setting, has emerged as a powerful means of ensuring the sustainability of a UHC system.^3^ However, with the multitude of challenges facing LMICs, such as weak institutions, limited human resource capacity, and funding challenges, institutionalizing HTA becomes a daunting task.

The experiences from LMICs, such as Thailand, India, China, Brazil, and so on, that have established some form of HTA process^4^, ^5^, ^6^ offer lessons on common challenges across settings. Countries that are implementing or planning to implement a systematic process for HTA can benefit from knowledge sharing and collaboration to a solution to what, alone, appear to be unsolvable challenges. In this article, we seek to identify the main challenges encountered by LMICs in Asia and Africa in institutionalizing HTA and potential solutions based on the experiences of those LMICs. We do so by drawing on a session hosted at the annual HTAsiaLink Conference in 2019, a unique platform for member countries to expand engagement in mutual learning and sharing solutions for institutionalising HTA.^7^

## Methods

The approach for this study involved identifying the challenges faced by countries through a survey, organizing a preconference session at the annual HTAsiaLink Conference in 2019 to discuss selected country experiences and a literature review. First, the focal HTA agencies from 8 countries (Australia, Taiwan, Republic of Korea, Japan, China, Malaysia, Singapore, and Thailand) of the HTAsiaLink Board identified 3 main challenges by responding to a survey conducted before the preconference session: financing of HTA work and organizations, building and retaining technical HTA capacity, and linking research to policy and stakeholder buy-in. Second, 4 specific countries (Bhutan, Kenya, Thailand, and Zambia) were selected to share their experiences around the challenges during the preconference session. These countries were selected because they have decided to institutionalize HTA as part of their priority setting process and are at different stages of institutionalization, offering varied lessons. Third, a total of 35 participants from 18 HTA organizations and 14 countries shared their experiences surrounding those challenges through a moderated discussion and a World Café. Fourth, a literature review was conducted on HTA to verify or fill-in information gaps from the data collected during the preconference session.

## Results: Challenges and Potential Solutions

### Challenge 1: Financing of HTA Work and Organization

*Financing HTA* in this article refers to the financial requirements for HTA organizations to conduct research, including research design, data collection, and analysis, and consult stakeholders or users to ensure that studies are in line with policy questions and are of acceptable quality. This entails using public or private sources for conducting the research or critically appraising evidence submitted by manufacturers or other HTA agencies.

Evidence-informed priority setting often occurs in an ad-hoc, one-off manner in LMICs without attention to the long-term, fixed budgetary support arrangements.^8^ Policymakers have not prioritized the need for consistent financing to sustain HTA institutions and conduct related activities. This could partly be explained by the fact that there may be limited political will from the Ministry of Finance or budget holders in the Ministry of Health, or that they are not fully aware of the value of investing in HTA. As a result, often, LMICs do not have a fixed line budget for HTA. As policy advisors, HTA units require sufficient and regular budgets for priority-setting work to be successfully conducted. In high-income countries, including Germany, Australia, and the United Kingdom, these numbers rise up to USD 90 million and USD 650 000, respectively.^8^ There may be different models for raising funds, and in Thailand, for example, the Health Intervention and Technology Assessment Program (HITAP) receives funds from users of HTA to conduct each assessment. Participants of the preconference called on governments of LMICs, including those that already decided to institutionalize HTA as part of their priority setting process, to establish sizable and fixed HTA budgets. In Bhutan, the nation’s main HTA body, the Essential Medicines and Technology Division under the Department of Medical Services, Ministry of Health, has been struggling to earmark budgets for HTA, which may be, at least partially, attributed to a lack of knowledge of HTA among staff in both the Ministry of Health and the Ministry of Finance, the latter being the one that authorizes annual budget allocation.

Moreover, HTA financing needs compete for already scarce resources assigned to policy research, not to mention the health sector. For example, in Kenya, 6.7% of government spending is currently allocated to health as a share of total government expenditure, which is relatively low in comparison to the Abuja declaration of 15%.^9^ In addition, the Kenyan government has historically not given research funding a priority. Although Kenya committed to allocating 2% of its GDP to research, it has so far only allocated an average of 0.7% of its GDP to all types of research across sectors, including health policy research.^10^ Such underfunding of research in LMICs cascades to the health sector where HTA research is neglected with insufficient, ad-hoc budgets, most of which come from external research funding organizations and donors in the short to medium term.^11^ In Zambia, although there is a small budget for research within the Ministry of Health, it is crowded-out by other areas and as a result, most priority setting work is mainly supported by partners such as the World Health Organization, USAID, Swedish government, etc.^12^ In particular, the Swedish government supported previous costing of a basic benefit package and the cost-effectiveness of an intervention for malaria, which contributed to changing the first line treatment.^13^

Funding for HTA work is limited and fragmented in most of LMICs, and chiefly comes from external and non-governmental funding sources. It is crucial to manage potential risks from different motives and agenda of these sources, including private sector, donors, and developmental partners, while deterring LMICs’ overreliance on them. In Bhutan, policymakers often do not have enough knowledge or internal capacity to assess the feasibility, and budget impact of implementing health interventions, especially when funded by external donors. The importance of HTA is realized when donors are about to phase out and the issue of sustainability of vertical programs is raised. To maintain such initiatives in LMICs, therefore, securing internal finances for HTA is needed.

For potential solutions to address insufficient HTA financing, the participants maintained that LMICs need policy initiatives for setting up a strong legal framework of evidence-informed priority setting that mandates HTA work with accountability. With UHC becoming a priority for several countries, HTA has garnered substantial attention, which is a good start. However, securing funds from domestic resources remains a challenge. One way of moving forward in this scenario would be to promote the value of HTA (and of HTA agencies) by generating evidence on the return on investment as well as economic and health impact of HTA, geared towards policymakers. Such efforts should aim to highlight that while HTA agencies require financial support, they offer an efficient value-for-money policy tool and can therefore be cost-saving. A recent simulation study has found that the work of HTA agencies is highly correlated with a decrease of health system costs and improvement of health benefits compared with the alternative of funding health interventions on a pure first-come-first-serve basis.^14^ Thus, HTA work and agencies deserve stable financial investment, including seed funding from policymakers. For additional funding, HTA agencies can enhance the ownership of policymakers through effective communication skills/materials for awareness raising and knowledge sharing of HTA among policymakers. They can also showcase the impact of HTA through pilot studies for a broad range of audiences such as potential funders (for example, all relevant ministries and agencies, not just ministry of health). To effectively work with industry as funders, participants recommended exploring the possibility for industry to underwrite the assessment of technologies or to create a research fund for donor-driven interventions in LMICs. In Zambia, the policymakers can look into the possibility of tapping into resources from mining factories and banks by leveraging the Public Private Partnership agenda highlighted in the national health strategic plan and the national health financing strategy. This is because the workers in these industries are quite often diagnosed with Tuberculosis (TB)/HIV and Non-communicable Diseases (NCDs), respectively; thus, such work would be directly relevant to their internal constituents. However, industry should maintain a distance throughout the process of research, namely, during agenda setting, financial feasibility, and sustainability of implementing the recommendations.

### Challenge 2: Building and Retaining Technical Capacity

This challenge refers to building, recruiting, and retaining relevant HTA personnel for the research (for example, clinical specialists, economists), operational (for example, administrative, legal), knowledge management (for example, Monitoring and Evaluation or M&E, knowledge sharing and dissemination), communications, and other aspects of HTA work.

The selection of the specific areas and number of staff largely depends on the legal mandate of the organization and available resources. Overall, the health system at all levels needs institutional capacity building for the successful translation of research into policy.^15^ As prerequisites to both, establishing HTA organizations and continuously pursuing advanced HTA, building and retaining technical capacity is a success factor for HTA organizations. Arguably, countries with nascent health systems face the challenge of limited human resource with very few professionally trained individuals competent in HTA in areas such as clinical effectiveness and economic assessments. Many countries struggle to train and retain such talent, although these are recognized as being critical in conducting HTA. For example, Kenya’s limited human resource capacity for HTA is concentrated in a few research/academic institutions.

All capacity building activities ultimately involve empowering individuals.^15^ Capacity building interventions can take a variety of forms, including providing technical assistance, in-depth consultations, virtual and in-person training sessions, online learning options, guidance materials in the form of knowledge products, and skills-based courses among others such as coaching and mentoring.^16^, ^17^, ^18^ A brief review of literature reveals that various models, theories, and frameworks for capacity development are used in public health^18^; the most applicable to the context of HTA is the United Nations Development Programme’s Individual, Node, Network, and Enabling Environment model.^19^ This model suggests that the approach to capacity building should be based on 4 principles. First, any capacity building approach should be closely aligned with the present and the future needs of the health system, organization, and individual. Second, incentives to learn and apply newly acquired skills should be in place. Third, the main aim of knowledge transfer should be to develop local personnel to ensure sustainability of capacity within country systems. Finally, efforts should be integrated with the existing system to strengthen and sustain the capacity at the national and local levels.^15^^,^^19^^,^^20^

Owing to the multidisciplinary nature of HTA, organizations face the constant need to hire and train talent from diverse academic and professional fields. Technical personnel from LMICs have limited opportunities for formal training programs (master’s and doctoral programs) and on-the-job trainings to build adequate capacity for advanced HTA work.^21^ The equally scarce opportunities in informal trainings contribute to the overall shortage of qualified HTA workforce in LMICs. They also face the increasing challenge of retaining trained professionals, who are likely to take jobs in industry and developmental organizations for better remuneration.^5^^,^^21^^,^^22^ Local universities are also not fully involved despite having principal HTA competencies in clinical, economic, communications, and other fields due to limited funding.

Participants at the preconference session identified 3 main challenges that hinder development of capacity within a public health system: lack of job security in HTA, need for public appreciation and incentives (both monetary and nonmonetary), and missing human resources (HR) mechanisms for career/skills progression. First, the job market in HTA tends to be unstable with much employee turnover. Postitions are oftentimes temporary (consultants, interns, and contract workers) and there is uncertainty of staff being promoted to a regular position. These factors put the HTA job market in a vicious cycle. Also, there are not many agencies specializing in HTA that are embedded within the health system, while universities in general lack adequate funding. To address these issues, HTA agencies should ensure hiring after training, coupled with a conducive working environment which may include opportunities to publish in journals and participate in regional/global conferences.

LMICs should also create a core HTA agency under the government with assigned budget, hiring power, and mandate, which can spearhead collaboration with international partners. HITAP of Thailand, for example, which, under the aegis of the International Decision Support Initiative (iDSI) and the Access and Delivery Partnership, seeks to develop HTA capacity of local governments, as is the case in Bhutan, Kenya, Thailand, and Zambia. Such collaboration can build local capacity to support both, HTA work and establishing a central HTA unit, eventually producing regular jobs. Capacity building at local universities is equally important. For example, there is a potential for establishing an HTA unit within the University of Zambia, which has the advantage of having an already competent cadre of HTA researchers and the ability to identify and train additional future HTA workforce. Second, due to the sporadic demand of HTA in LMICs and limited understanding of this relatively nascent field, HTA personnel receive little public appreciation and their salaries and benefits are relatively low compared with other areas of public health research (such as epidemiological studies, maternal health studies, or even health financing studies). To address these issues, LMICs should ensure that HTA personnel have satisfactory incentives, a decision backed by official laws or provisions and acknowledged by high-level policymakers. HTA organizations can disseminate effective communications materials to raise awareness on the importance of HTA among the targeted policymakers who are in a position to guarantee such incentives and express public appreciation for HTA. Furthermore, HTA organizations in LMICs generally implement HR policies and governance structures that may inadvertently cause a high employee turnover rate. In Zambia, one key challenge that has been identified by the National Health Strategic Plan is the lack of research positions, including those for HTA, within the Ministry of Health. Although the ministry may not need to conduct HTA, it is recommended that the ministry nevertheless strengthen its in-house technical HTA knowledge and expertise to ensure effective oversight of HTA activities nationwide. The ministry has transformed its research unit into a research authority, while its role is mostly about regulating research. LMICs should utilize a government research unit to promote the idea of enhancing HTA capacity within both the ministry and a central HTA agency. HTA organizations’ HR structure should also be revised favorably to encourage innovation in managing interdisciplinary works of HTA with a focus on enhancing the knowledge of the HTA-related workforce. This may include preventing HTA researchers from being rotated to non-HTA departments, as in Bhutan where staff are transferred every 5 years.

### Challenge 3: Linking Research to Policy and Stakeholders Buy-In

This challenge refers to using evidence for policymaking which implies that well-designed, and well-conducted HTA research, with consideration of the local context, should guide policymaking for healthcare priority setting.

Inclusion of stakeholders in the process of topic selection, evidence generation, and funding recommendations is critical to ensuring relevance of HTA to country priorities in any country regardless of income level.^6^ The examples of stakeholders include policymakers, ministry/regulatory agencies, medical associations, manufacturers, pharmaceutical industry, hospitals, patient groups, media, academia, civil society, and the public. Even with emerging evidence which suggests that HTA in LMICs, while limited, is increasing and momentum is building particularly in the areas of capacity building and research tool development, the results of HTA studies are not being used systematically to inform national level decision making. In other words, the low translation rate of HTA hinders informing regulatory, coverage/formulary and reimbursement decisions in LMICs. Instead, they are often conducted based on the interest of specific groups and not to enhance macro-level public policy goals or the overall health system.^23^ For instance, in Zambia, although relevant government units conduct studies using their research budgets, such work is normally restricted to traditional activities which only may be loosely classified as HTA (for example, planning, budgeting, resource tracking, and supporting activities of national formulary committees).

Building consensus among stakeholders becomes crucial as the diversity of HTA topics and multiple interests have a bearing on the HTA process. From research to actual implementation as a policy option, HTA by its nature involves a number of stakeholders with competing agendas, which need to be compromised and reflected throughout the process. Utilization of research findings in policymaking is also not straightforward, owing to some common barriers that influence the functioning of a health system. The barriers of getting HTA research into practice with effective stakeholder buy-in are multifaceted, ranging from the lack of available local studies of adequate quality, lack of understanding (and thus trust) of HTA among stakeholders, to organizational allegiances or institutionalized modes of thinking and behavior, and political pressures on policymakers. It is important to consider to what extent these barriers in healthcare infrastructure, institutions, policy processes, and political pressures are distinctive or similar to other LMICs.^24^

To tackle most of these barriers, the participants at the preconference session suggested that HTA agencies which lack strategic communication capacity can focus on strengthening collaborative efforts among stakeholders to find and institutionalize possible ways to translate HTA research into policy. This is because even with quality HTA studies, stakeholders, including policymakers, require good understanding of the research results. Suppliers of evidence should thus be effective communicators with each stakeholder, which sees the world from the perspective of their audience and understands the policy process differently.^25^ Even though it would be preferred if an agency with legal mandate can facilitate these efforts with a sense of ownership, the role of HTA champions, within or outside these agencies, is also important in communicating effectively with different stakeholders. The participants pointed out that the consistent (or even growing) knowledge gap between HTA researchers and policymakers causes priority setting practices in LMICs to keep operating in silos. For example, in Kenya the National Health Insurance Fund develops benefit packages that are not aligned with the ones developed by the Ministry of Health. Although research organizations carry out economic evaluations and other analyses, these are often not at the request of the Ministry of Health and the findings of these studies are not always used to inform policymaking. Without attempting to build a certain level of HTA knowledge in a harmonious way, policymakers cannot properly implement the HTA research results into actual policy in the context of the national health system. In addition, publishing guidelines for conducting HTA can facilitate reporting the results in a standardised manner and allow policymakers to compare analyses in an efficient manner, enabling them to better interpret results and compare studies.^26^

For potential solutions to improve strategic communication skills, participants suggested the use of policy briefs to share HTA results and providing awareness raising opportunities to stakeholders in LMICs. In Zambia, the National Health Research Authority (NHRA) currently receives support from the Bloomberg Foundation to improve its policy briefs on economic evaluations studies. The country has also created new communication platforms where the newly established Knowledge Translation Technical Working Group (TWG) under the NHRA works to improve the uptake of research into policy along with other TWGs within Ministry of Health (for example, the Health Financing TWG and the Vaccination TWG). They are composed of a multidisciplinary team from the government, cooperating partners, and the University of Zambia. The knowledge translation function of the NHRA is spelt out on the National Health Research Act, as the legal framework is crucial for the NHRA to properly execute its functions.^27^ This helps policymakers become more confident in using and interpreting HTA evidence and explaining it to others. The participants agreed that having designated communication staff who can strategize and enhance dissemination efforts through effective translation of HTA studies and easy phrasing of research results will assist policymakers in utilising the results of HTA studies. Another way is to provide policymakers with awareness raising consultations as part of capacity building on the potential benefits of HTA-generated evidence to inform policymaking. In Zambia, health researchers use dedicated funding from SIDA to improve dialogue between them and policymakers as a means to strengthening health economics research.^13^ The National Health Financing Strategic Plan mentions University of Zambia as an organization that should support the Ministry of Health on health financing research including HTA.^28^ Under the funding, researchers can raise awareness of HTA among the ministry officials by jointly setting the research agenda with policymakers, conducting economic evaluations, and presenting their results to them. The participants also urged that HTA champions with certain expertise around health program planning, operations, or legislature should lead the implementation of research to the actual policy, while managing stakeholders’ expectations and demands. LMICs should identify such champions and assign much needed financial, regulatory, and legal authority to function as the facilitator between HTA researchers, policymakers, and other stakeholders. In Kenya, the government established 2 expert panels under the Ministry of Health (the Health Benefits Package Advisory Panel and the National Health Insurance Fund Health Financing Reforms Expert Panel) as focal points to use evidence for stakeholder buy-in.^17^ The institutional arrangements in LMICs should support these facilitators for a credible process to incorporate evidence into policy transparently and through participation. Under such a framework, stakeholders can jointly identify impactful topics for HTA while ensuring that assessments are not driven by supply but rather by need and demand.^29^

## Conclusions

It is commonly acknowledged that the missing links for successfully institutionalizing HTA is the lack of political commitment, funding availability, adequate technical capacity, and of inclusive stakeholders buy-in.^30^, ^31^, ^32^ The participants of the preconference on South-South Knowledge Exchange of HTA not only reiterated these challebes but also showed that they are closely interlinked. Thus, any potential solution should take into account all of the challenges with consideration of the local context. Equally important is that a national HTA agency with a with mandate should consistently strive to improve policymakers’ awareness on the importance of HTA through active and targeted dissemination efforts.

By bringing together policymakers and technical experts from LMICs that share certain commonalities, such as the level of economic and social development and similar challenges within the health system, South-South platforms provide a conducive environment for experiential learning and exchanges of ideas, experiences, and expertise. South-South platforms are thus a valuable source of knowledge and an essential element of sustainable development^9^ because they promote the collective determination of solutions that are more likely to be appropriate, feasible, and implementable within the specific contexts of LMICs.^15^ Such a South-South platform can also provide a mechanism through which countries can actively facilitate technical and capacity building support and cooperate on implementing an initiative such as HTA.

This study benefitted from the HTAsiaLink conference, which is an effective knowledge exchange platform. The participants in this preconference session hailed from various countries and backgrounds were able to provide inputs based on their in-country experience, and included, representatives from LMICs who were program managers, academics and policymakers. Furthermore, the findings from the session have been corroborated by a literature review. As a limitation, the authors acknowledge that the list of challenges for the institutionalization of HTA mentioned here is not an exhaustive one, and the challenges covered in this article reflect those from selected country settings.

## Article and Author Information

**Author Contributions:** *Concept and design*: Kim, Sharma, Teerawattananon, Oh, Ong, Dabak

*Acquisition of data*: Hangoma, Adhikari, Pempa, Kairu, Orangi

*Analysis and interpretation of data*: Kim, Sharma, Teerawattananon, Oh, Ong, Hangoma, Adhikari, Pempa, Kairu, Orangi, Dabak

*Drafting of the manuscript*: Kim, Sharma

*Critical revision of the paper for important intellectual content*: Oh, Ong, Hangoma, Adhikari, Pempa, Kairu, Orangi, Dabak

*Provision of study materials or patients*: Teerawattananon

*Supervision*: Teerawattananon

**Conflict of Interest Disclosures:** The authors reported no conflicts of interest.

**Funding/Support:** This work was supported by the Access Delivery Partnership, hosted by the 10.13039/100016195United Nations Development Programme, United States, with funding from the Government of Japan. The Health Intervention and Technology Assessment Program (HITAP) is funded by the Thailand Research Fund under a grant for Senior Research Scholar (RTA5980011). HITAP’s International Unit is supported by the Interantional Decision Support Initiative (iDSI) to provide technical assistance on health intervention and technology assessment to governments in low- and middle-income countries. iDSI is funded by the Bill & Melinda Gates Foundation [OPP1202541], the United Kingdom’s Department for International Development, and the Rockefeller Foundation.

**Role of the** **Funder/Sponsor:** The findings, interpretations, and conclusions expressed in this article do not necessarily reflect the views of the funding agencies.
